# Autophagy-mediated negative feedback attenuates the oncogenic activity of YAP in pancreatic cancer

**DOI:** 10.7150/ijbs.61795

**Published:** 2021-08-21

**Authors:** Ting Sun, Hui Peng, Wenhao Mao, Liwei Ma, Hongyang Liu, Jia Mai, Lin Jiao

**Affiliations:** 1 Department of Clinical Laboratory, The First Affiliated Hospital of Zhengzhou University, Zhengzhou 450052, China.; 2 Department of Clinical Oncology, the First Affiliated Hospital of Zhengzhou University, Zhengzhou 450052, China.; 3 Department of Obstetrics and Gynecology, The Third Affiliated Hospital of Zhengzhou University, Zhengzhou 450052, China.; 4 Department of Laboratory Medicine, West China Second Hospital, Sichuan University, Chengdu 610041, China.; 5 Department of Laboratory Medicine, West China Hospital, Sichuan University, Chengdu 610041, China.

**Keywords:** pancreatic cancer, YAP, autophagy, Atg5, verteporfin

## Abstract

Pancreatic ductal adenocarcinoma (PDAC) is the most lethal malignancy in humans, and new therapeutic targets are urgently needed. Yes-associated protein (YAP) plays a significant role in cancer progression. Autophagy is also closely associated with various human cancers. However, the interplay between YAP and autophagy in PDAC remains poorly understood. In this study, we found that YAP was upregulated and activated in PDAC. Further analysis revealed that there is a YAP-autophagy feedback loop in pancreatic cancer. Mechanistically, YAP activates autophagy by promoting Atg5 transcription via TEAD1-mediated binding, while autophagy negatively regulates YAP through autophagic degradation. The hyperactivation of YAP in PDAC unbalances the YAP-autophagy circuit and promotes cancer progression. Inhibition of autophagy enhances the oncogenic activity of YAP in PDAC. The autophagy activator rapamycin promotes the antitumor effect of verteporfin, a YAP inhibitor. Therefore, our study elucidated the interaction between YAP and autophagy in PDAC and our results suggest that targeting the YAP-autophagy circuit may be a new therapeutic strategy for pancreatic cancer.

## Introduction

Pancreatic cancer, mostly pancreatic ductal adenocarcinoma (PDAC), is the most lethal malignancy in humans, with a 5-year survival rate of approximately 9% and a median survival of approximately 6 months 1-3. The high mortality of pancreatic cancer patients is mainly due to the difficulty in diagnosing the disease at the early stage, aggressive local invasion and easy metastasis 4. Currently, gemcitabine, the first-line chemotherapeutic drug for pancreatic cancer, provides only a limited survival advantage in PDAC patients, and the therapeutic efficacy is unsatisfactory 5. Activating mutations in KRAS are the most frequent genetic events and are present in the majority of pancreatic cancer patients 6. Experiments conducted on genetically engineered mouse models have also substantiated the critical role of KRAS mutations in pancreatic cancer initiation and progression 7-10. Unfortunately, KRAS has been proven to be difficult to target and the therapeutic strategy of directly blocking KRAS activity with small-molecule inhibitors has proven challenging 11. Therefore, it is of substantial importance to elucidate the underlying mechanism of PDAC and develop new therapeutic strategies.

Studies have shown that Yes-associated protein (YAP), the major nuclear effector in the Hippo signaling pathway, enables the bypass of oncogenic KRAS addiction in pancreatic cancer 12. Moreover, as a critical oncogenic KRAS effector, YAP is essential for neoplastic progression to PDAC 13. In mammals, the Hippo pathway plays a critical role in organ size control, tissue homeostasis and stemness by controlling cell proliferation and death 14-17. When the Hippo pathway is activated, MST1 and MST2 kinases phosphorylate and activate LATS1/2 kinases, which in turn phosphorylate YAP and TAZ, leading to their cytoplasmic retention or degradation 18. When Hippo signaling is inhibited, hypophosphorylated YAP/TAZ is translocated into the nucleus where it mainly interacts with TEAD transcription factors acting as coactivators of the transcription of target genes 19, 20. Accumulating evidence has suggested that dysregulation of the Hippo pathway plays a crucial role in cancer progression 21. YAP has been confirmed to be involved in several human cancers 17, including breast cancer 22, lung cancer 23, ovarian cancer 24 and liver cancer 25. In pancreatic cancer, YAP contributes to cell proliferation and invasion and inhibiting YAP expression suppresses pancreatic cancer progression 26. Moreover, YAP is an independent prognostic marker in patients with PDAC and associated with liver metastasis 27. Despite these research advances, the precise mechanisms underlying YAP dysregulation and the possibility of YAP-targeted therapy for pancreatic cancer remain to be explored.

Macroautophagy (hereafter autophagy) is an evolutionarily conserved lysosome-dependent cellular catabolic degradation pathway 28. Autophagy plays a dual role in cancer. In the early stages of tumorigenesis, autophagy can clear damaged organelles and toxic unfolded proteins to maintain genomic stability, thereby inhibiting malignant transformation. Conversely, after tumor formation, increased autophagic flux contributes to the survival and growth of tumor cells, and promotes tumor invasion and metastasis under various environmental pressures 29, 30. This makes autophagy an attractive target for cancer therapy.

Recent studies have indicated that the Hippo-YAP pathway is involved in the regulation of autophagy 31. Maejima et al. showed that MST1 inhibits autophagy by promoting the interaction between Beclin1 and Bcl2, suggesting a role for the Hippo pathway in integrating autophagy and apoptosis during cellular stress 32. Another study revealed that the Hippo kinases STK3/STK4 promote autophagy via direct phosphorylation of LC3 33. Moreover, autophagy is regulated by YAP/TAZ. Although YAP/TAZ controls autophagic flux by regulating the degradation of autophagosomes, YAP/TAZ is also essential for the maturation of autophagosomes into autolysosomes 34, 35. Conversely, YAP is an autophagy substrate and YAP dysregulation is associated with hepatocarcinogenesis in autophagy-deficient livers 36. Collectively, these studies suggest that there is crosstalk between YAP and autophagy, but how they are involved in pancreatic cancer progression remains unclear.

In the present study, we found that YAP was overexpressed and activated in PDAC and that there was a YAP-autophagy feedback loop. YAP activated autophagy by promoting Atg5 transcription, while autophagy negatively regulated YAP through autophagic degradation. Aberrant hyperactivation of YAP in PDAC led to an imbalanced YAP-autophagy circuit. Autophagy weakened the oncogenic activity of YAP in PDAC, and autophagy activation induced by rapamycin promoted the antitumor effect of verteporfin, an inhibitor of YAP, suggesting that the YAP-autophagy circuit is a potential therapeutic target for pancreatic cancer.

## Materials and methods

### Cell lines, culture conditions and reagents

Pancreatic cancer cell lines (AsPC-1, BxPC-3, PANC-1, CFPAC-1 and SW1990) were obtained from the Cell Bank of Type Culture Collection of Chinese Academy of Sciences (Shanghai, China) and maintained in Dulbecco's modified Eagle's medium (DMEM) supplemented with 10% fetal bovine serum (Gibco, Grand Island, NY, USA). The cells were incubated at 37 °C in a humidified atmosphere with 5% CO_2_. Antibodies against YAP (#14074) and ATG5 (#2630) were purchased from Cell Signaling Technology (Danvers, MA, USA). Anti-active YAP (ab205270) antibody was purchased from Abcam (Cambridge, MA, USA). Antibodies against p62 (sc-28359) and GAPDH (sc-32233) were purchased from Santa Cruz Biotechnology (Dallas, TX, USA). Anti-LC3 (NB100-2220) antibody was obtained from Novus Biologicals (Littleton, CO, USA). Anti-Flag antibody (F3165), cycloheximide (CHX, 01810), verteporfin (SML0534) and chloroquine (CQ, C6628) were obtained from Sigma-Aldrich (St.Louis, MO, USA).

### RNA isolation and RT-qPCR

Total RNA was isolated using the Total RNA Kit I (Omega Biotech, Doraville, GA, USA) according to the manufacturer's instructions. For RT-qPCR, RNA was reverse transcribed to cDNA by using the PrimeScript™ RT reagent kit (Takara, Dalian, China). The cDNA was amplified on a 7500 Real-Time PCR System using SYBR Green Master Mix (Roche, Basel, Switzerland). All samples were normalized to GAPDH. The primer sequences are listed in supplementary Table S1.

### Cell proliferation assays

Cell viability was detected by CCK-8 and EdU assays. Cells were seeded in 96-well plates (4,000 cells/well), incubated overnight for attachment and then treated with indicated agents for different lengths of times. The medium was replaced with CCK-8 at 37 °C for 2 hours, and the absorbance at 450 nm was measured with a microplate reader (BIO-RAD, Hercules, CA, USA). EdU assays were performed using the EdU Cell Proliferation Assay Kit (Ruibo, Guangzhou, China) according to the manufacturer's instructions. All experiments were performed in triplicate.

### Wound-healing assay

AsPC-1 and BxPC-3 cells were seeded in 12-well plates and grown to 90% confluence. Then, scratch wounds were generated by using a plastic pipette tip, which was recorded as the 0 h. Then, the scratch was imaged at 24 h or 48 h. Cell migration was assessed by measuring the movement of cells into the scratch wounds.

### Transwell invasion assay

Matrigel-coated invasion assays were performed using a 24-well Transwell chamber system (Corning, NY, USA) as previously described 37. Briefly, 5×10^4^ cells in 400 μL serum-free culture medium were placed into the upper chamber, which was coated with Matrigel (BD, New Jersey, USA). A total of 600 μL medium supplemented with 20% FBS was added to the lower chamber. After incubation for 24 h, cells were fixed in 4% paraformaldehyde and stained with 0.1% crystal violet (Sigma-Aldrich, St. Louis, MO, USA) for 30 min. The stained cells were analyzed.

### ChIP analysis

ChIP assays were carried out using the EZ-ChIP kit (Millipore, Massachusetts, USA) according to the manufacturer's instructions using an antibody against YAP. Briefly, cells were lysed and then sonicated to obtain DNA fragments (500-800 bp). Next, samples were immunoprecipitated overnight at 4 °C with YAP antibody, supplemented with magna ChIP™ protein A/G beads. After washing, elution and decrosslinking, the quantity of immunoprecipitated DNA was analyzed by RT-qPCR using the indicated primers listed in supplementary Table S2.

### Luciferase reporter assay

293T cells were seeded in 12-well plates and transfected with the pGL3-Atg5 (wild type or mutated TEAD1-binding site) promoter reporter plasmid, pcDNA3.1-Flag-YAP or an empty vector, and a Renilla luciferase vector for normalization. Relative luciferase activity was measured with the Dual-luciferase Reporter Assay System (Promega, Madison, WI, USA).

### Plasmid constructs and transfection

A plasmid encoding human YAP was cloned into the pcDNA3.1 vector with a Flag tag. For transient transfection, plasmids were pretransfected with Lipofectamine 2000 (Invitrogen, Carlsbad, CA, USA) for 24 hours and then processed with the indicated treatment as described. siRNAs against YAP were produced by GenePharma (Suzhou, China) and transfected using Lipofectamine RNAiMAX Transfection Reagent (Invitrogen, Carlsbad, CA, USA). The target sequence for siYAP#1 was 5'- GGAAGCTGCCCGACTCCTTCT-3'; the target sequence for siYAP#2 was 5'- GCAGGTTGGGAGATGGCAAAG-3'. For stable YAP knockdown, pLKO.1-shYAP#1 (27368) and pLKO.1-shYAP#2 (27369) were obtained from Addgene. For stable Atg5 knockdown, the Atg5 shRNA plasmid (sc-41445-V) was obtained from Santa Cruz Biotechnology (Dallas, TX, USA). Stable cell lines were established as previously described 37.

### Tissue microarray slides and immunohistochemistry (IHC)

*In vivo* active YAP expression was detected by IHC using tissue microarrays (PA2081a, AlenaBio, Xi'an, China). The tissues were incubated with primary anti-active-YAP antibody (1:100, ab205270, Abcam) and biotin-conjugated secondary antibody. Hematoxylin was used as the counterstain. Immunostaining degree of each sample was evaluated independently by two pathologists based on nuclear staining intensity (0, negative; 1, weak; 2, moderate; 3, strong) and the percentage of positive cells (0, <5% positive cancer cells; 1, 6-25% positive cancer cells; 2, 26-50% positive cancer cells; 3, 51-75% positive cancer cells; 4, ≥76% positive cancer cells). The final immunoreactivity score was calculated as the product of the intensity score and the extent score.

### Cycloheximide (CHX) chase assay

AsPC-1 cells were incubated at 90% confluency in complete growth media supplemented with the protein synthesis inhibitor CHX (50 μM) or vehicle. Cells were collected in RIPA buffer with proteinase inhibitors at 0, 4, 8, 16 and 24 h after incubation. The lysate was sonicated and centrifuged for 10 min at 12,000g, and the resulting supernatants were analyzed by immunoblotting.

### Immunofluorescence analysis

For immunofluorescence analysis, cells were plated in chamber slides, fixed in methanol for 10 min at room temperature, and permeabilized with 5% bovine serum albumin (BSA) in PBST. Cells were then exposed to primary antibodies (anti-YAP 1:200) diluted in PBST with 5% BSA overnight at 4 °C. After washing three times with PBS, secondary antibody (Alexa Fluor 488 goat anti-rabbit 1:200) diluted in PBST was added and the slides were incubated for 1 h at room temperature. Cells were then washed in PBS and mounted using 4,6-diamidino-2-phenylindole (DAPI) to counterstain DNA. Images were collected using a confocal microscope (Olympus FV-1000).

### Autophagy analysis

Autophagy was measured by quantitation of GFP-LC3 puncta using fluorescence microscopy as previously described 28. Cells were infected with appropriate concentrations of lentivirus carrying GFP-LC3 to express a close-to-endogenous level of GFP-LC3. After treatment, cells were fixed with 4% paraformaldehyde for 20 min and rinsed with PBS twice. The total number of cells in the images was determined by nuclear staining with 4,6-diamidino-2-phenylindole. Cells were mounted and visualized under a confocal microscope (Olympus FV-1000).

### Xenograft tumor-formation assay and therapeutic treatment

Female BALB/C nude mice at 4-5 weeks of age were obtained from the Beijing Vital River Laboratory Animal Technology Co., China. A total of 5×10^6^ AsPC1 cells were subcutaneously inoculated into the right flank of mice to establish pancreatic cancer xenografts. Approximately 8 days after subcutaneous implantation, the mice were randomly divided into four groups and administered verteporfin (50 mg/kg/day), rapamycin (4 mg/kg/day), verteporfin plus rapamycin, or PBS as a control. During the treatment, tumor volume was measured every 4 days and calculated using the following formula: length × width^2^ × π/6. Mice with tumor implants were euthanized 32 days after drug treatment, and the tumor xenografts were excised and weighed.

### Statistical analysis

GraphPad Prism software (Version 5.0) was used for experimental data analysis. All experiments were independently repeated at least three times. Student's t test was used to identify significance between groups, and statistical significance was determined when *p* < 0.05 (two-tailed). Values are expressed as the mean ± SEM.

## Results

### YAP is upregulated and hyperactivated in PDAC

**Figure 1:**
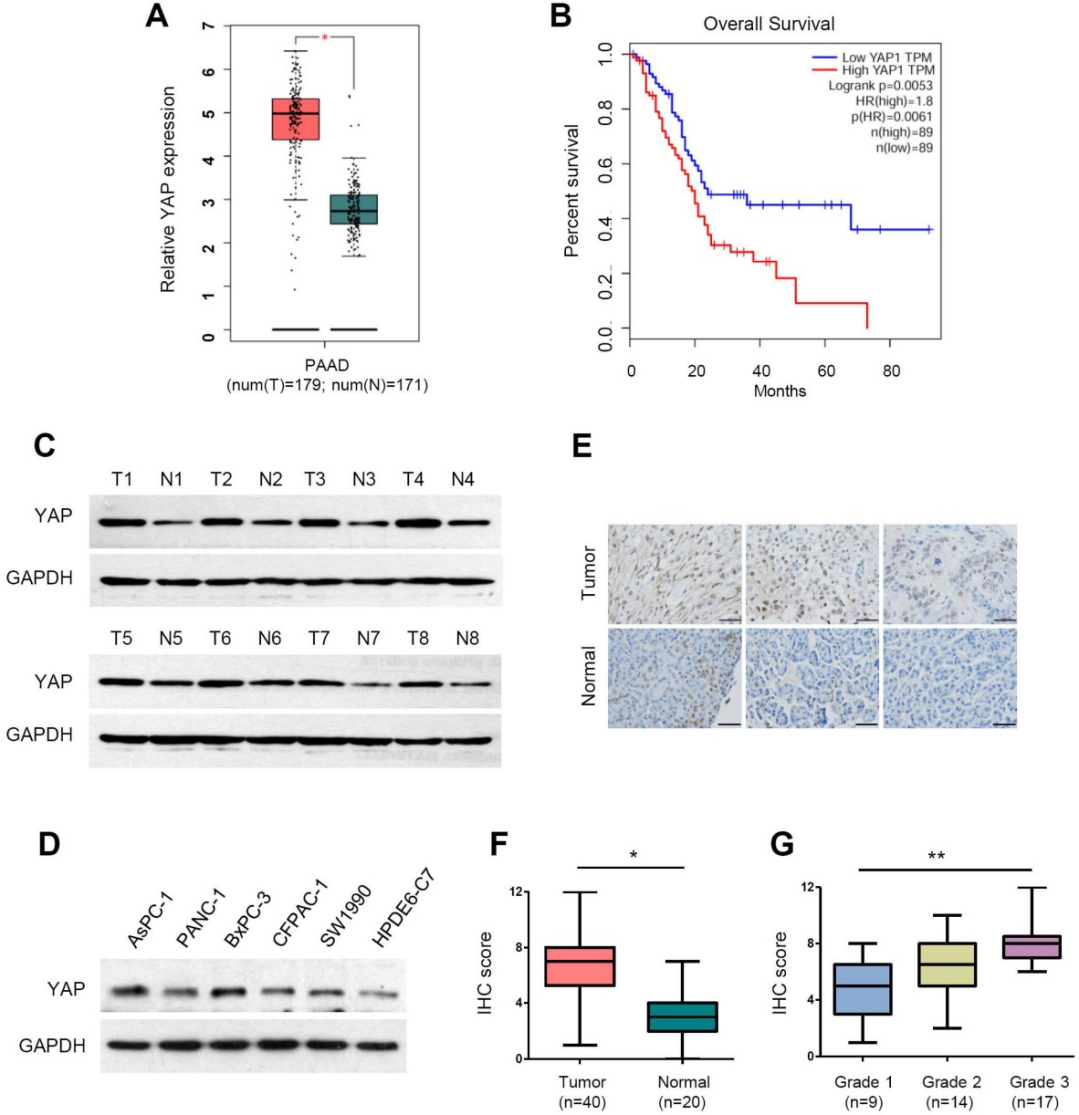
** The expression of YAP and its clinical significance in pancreatic cancer. (A)** The mRNA expression level of YAP in pancreatic cancer tissues (n=179) compared with that in normal tissues (n=171) from the TCGA and GTEx databases. **(B)** Kaplan-Meier analysis of YAP mRNA expression in pancreatic cancer based on the TCGA database. **(C)** Western blot analysis of YAP protein levels in eight pairs of clinical pancreatic cancer tissues (T) and adjacent normal tissues (N). **(D)** Western blot analysis of YAP protein expression in five human PDAC cell lines and HPDE6-C7 cells. **(E)** Representative immunohistochemistry (IHC) staining of active YAP in pancreatic cancer tissues and normal tissues. Scale bar, 50 µm. **(F)** The IHC scores of YAP expression levels in 40 pancreatic cancer tissues and 20 normal tissues. **(G)** The IHC scores of YAP expression from grade 1 to 3 pancreatic cancer specimens. The statistical analyses were performed by Student's *t* test (A, F), one-way ANOVA (G), and Kaplan-Meier analysis (B). *p<0.05, **p<0.01, ***p<0.001.

YAP has been reported to be overexpressed in pancreatic cancer 26, 38, 39. To validate the mRNA expression of YAP in PDAC, we analyzed the RNA-sequencing data from the TCGA and GTEx databases using the web-based tool GEPIA (Gene Expression Profiling Interactive Analysis, http://gepia.cancer-pku.cn/) 40. The analysis showed that YAP mRNA levels were significantly upregulated in PDAC tissues compared with normal pancreatic tissues (Figure 1A). Moreover, patients with high YAP expression had significantly worse overall survival rates than those with low YAP expression (Figure 1B). We conducted western blot analysis of YAP protein expression in eight pairs of clinical PDAC and adjacent normal tissues. Compared with that in adjacent normal tissues, YAP protein levels were upregulated at varying degrees (Figure 1C). Consistently, increased YAP protein expression was also detected in the five PDAC cell lines (AsPC-1, PANC-1, BxPC-3, CFPAC-1 and SW1990) compared with that in the immortalized human normal pancreatic duct epithelial cell line HPDE6-C7 (Figure 1D).

In total YAP protein, only dephosphorylated YAP can enter the cell nucleus to activate gene transcription, thus playing an oncogenic role. To accurately evaluate the level of activated YAP in PDAC tissues, we used an antibody that specifically recognizes the active (unphosphorylated) form of YAP. We then carried out immunohistochemical (IHC) staining for activated YAP in a human pancreatic cancer tissue microarray consisting of 40 PDAC and 20 normal pancreatic tissues (Figure 1E). The results showed that active YAP expression was significantly increased in PDAC tissues compared to that in normal tissues (Figure 1F). Moreover, we found that active YAP expression in PDAC correlated with histological grade, and the active YAP score in grade 1, grade 2 and grade 3 PDAC gradually increased (Figure 1G). These data indicate that YAP is overexpressed and hyperactivated in PDAC.

### YAP activates autophagy by promoting Atg5 transcription

**Figure 2:**
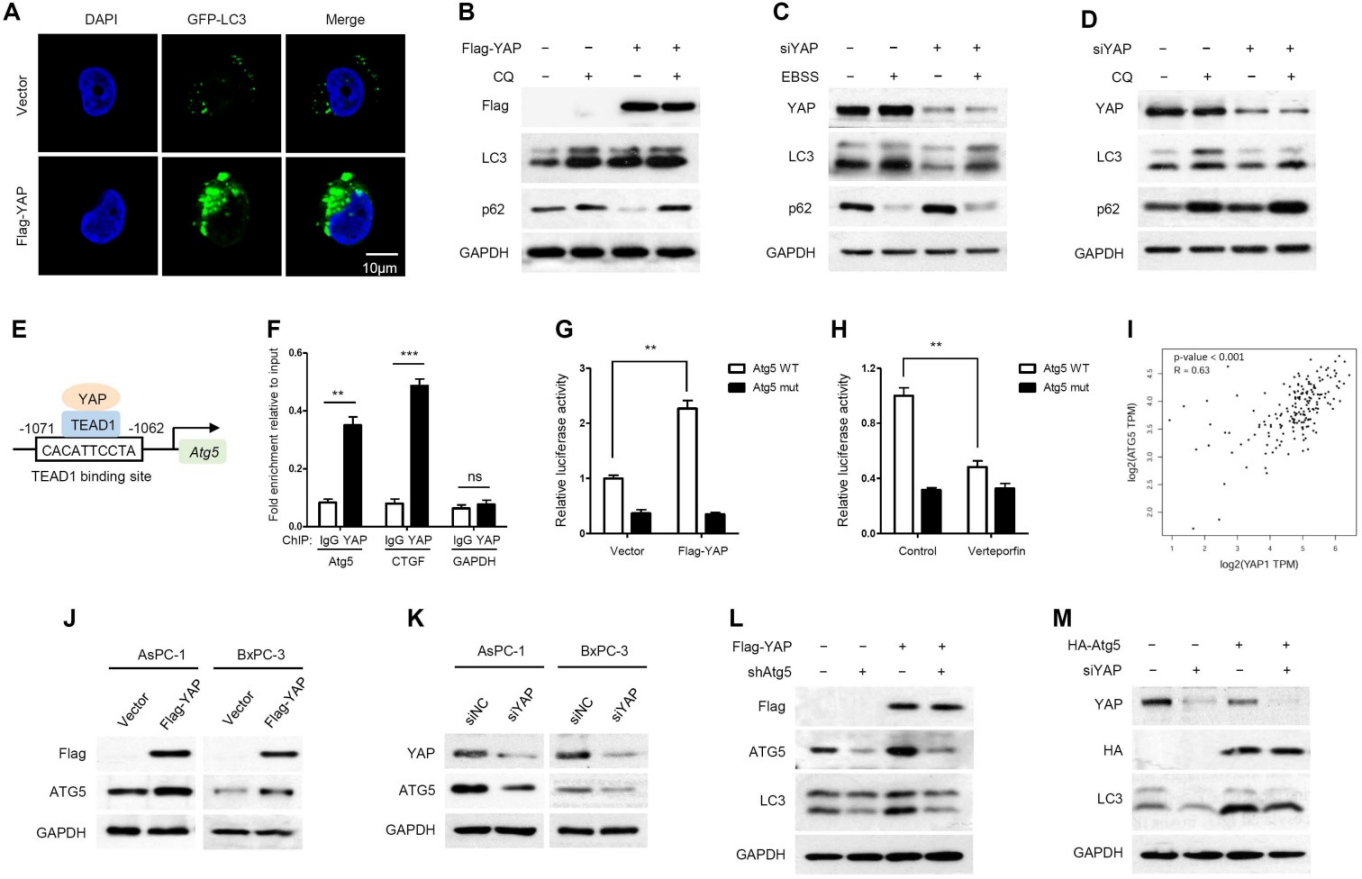
** YAP activates autophagy by promoting Atg5 transcription. (A)** Immunofluorescence analysis of GFP-LC3 in AsPC-1 cells transfected with Flag-YAP or a vector control. **(B)** AsPC-1 cells expressing either the vector or Flag-YAP were treated with or without CQ (100 mM) for 12 h, and protein expression was measured by immunoblotting. **(C)** AsPC-1 cells transfected with either the negative control or siYAP were treated with or without EBSS for 2 h, and protein expression was measured by immunoblotting. **(D)** AsPC-1 cells transfected with either the negative control or siYAP were treated with or without CQ (100 mM) for 12 h, and protein expression was measured by immunoblotting. **(E)** Predicted TEAD1-binding site in the promoter region of Atg5 based on the highest prediction score. **(F)** ChIP assays with YAP antibody or control IgG were performed in AsPC-1 cells. The binding of YAP to the Atg5 promoter was analyzed by RT-qPCR. CTGF and GAPDH were used as the positive and negative controls, respectively. **(G, H)** Luciferase reporter assays in 293T cells transfected with Atg5 promoter reporter containing wild-type (Atg5 WT) or mutated TEAD1-binding site (Atg5 mut) together with YAP overexpression or verteporfin (2 μM) treatment. **(I)** Correlation between YAP and ATG5 expression in the TCGA pancreatic cancer database. **(J, K)** Immunoblot analysis of YAP and ATG5 expression in AsPC-1 and BxPC-3 cells transfected with Flag-YAP or siYAP. **(L)** AsPC-1 cells were transfected with shAtg5 and Flag-YAP simultaneously or separately, and protein expression was measured by immunoblotting. **(M)** AsPC-1 cells were transfected with HA-Atg5 and siYAP simultaneously or separately, and protein expression was measured by immunoblotting. Data are shown as the mean ± SEM from three independent experiments. Student's *t* test. *p<0.05, **p<0.01, ***p<0.001.

It has been reported that YAP is involved in autophagy regulation 34, 35. We wondered whether YAP plays a role in regulating autophagy in PDAC. As shown in Figure 2A, GFP-LC3, a highly specific fluorescent marker of autophagosomes, was significantly increased as puncta in YAP-overexpressing AsPC-1 cells. To investigate whether the increased GFP-LC3 puncta observed upon YAP overexpression reflect increased autophagic flux or blocked autophagosome turnover, the effects of YAP were analyzed in the presence of chloroquine (CQ), an inhibitor of autophagosome degradation. The conversion of the soluble form of LC3 (LC3I) to the lipidated form (LC3II) is a sign of autophagy activation, and p62 is recognized as a substrate of autophagic degradation. We found that YAP overexpression plus CQ treatment had a synergistic effect in inducing the accumulation of LC3II, and CQ blocked YAP-induced p62 degradation (Figure 2B). These results suggest that YAP overexpression leads to an increase in autophagic flux. To complement these conclusions, we applied Earle's balanced salt solution (EBSS) to induce nutrient starvation and trigger autophagosome formation, which is the initial stage of autophagy. Indeed, YAP knockdown inhibited autophagy initiation. Even under nutrient starvation conditions, YAP depletion significantly inhibited autophagy activation (Figure 2C). Moreover, YAP depletion also reduced endogenous LC3 accumulation induced by CQ, but enhanced p62 accumulation (Figure 2D). While our findings indicate that YAP activates autophagy by inducing autophagosome formation, YAP has been reported to interfere with autophagic flux by enhancing autolysosome degradation in breast cancer cells 41, suggesting diverse, context-specific regulatory roles of YAP in autophagy.

The transcription factors TEAD1-4 mediate YAP-dependent gene expression even though they have been suggested to be functionally redundant 42. To establish which TEAD is essential for YAP-mediated transcriptional activity in PDAC cells, we assessed the activity of the TEAD-dependent luciferase reporter gene upon siRNA-mediated depletion of individual TEADs. TEAD1 depletion had a potent effect on reporter gene activity, while knockdown of TEAD2/3/4 had only marginal effects (Supplementary Figure S1).

Moreover, we used the JASPAR database 43 to analyze the promoter regions of several crucial autophagy-related genes, such as ULK1, Beclin1, Atg5 and Atg7. Notably, multiple TEAD1-binding sites were identified in the promoter region of Atg5 (Supplementary Table S3). The binding site with the highest prediction score in the promoter region of Atg5 was pursued as a candidate for detailed study (Figure 2E). In addition, to exclude the regulation of other autophagy-related genes by YAP/TEADs, the expression levels of ULK1, Beclin1, Atg5 and Atg7 were detected by RT-qPCR after YAP knockdown. YAP knockdown had a significant effect on Atg5 transcription but little effect on other autophagy-related genes (Supplementary Figure S2).

To investigate whether Atg5 is a direct target gene of YAP-TEAD1, we performed a chromatin immunoprecipitation (ChIP) assay, and CTGF was used as a positive control. YAP was recruited to the promoters of Atg5 and CTGF, but not to the negative control GAPDH gene (Figure 2F and Supplementary Figure S3). The competency of TEAD1 binding was further examined using a luciferase reporter assay. Verteporfin, an antagonist of the YAP-TEAD association, was applied to inhibit YAP transcriptional activity 44. YAP overexpression and verteporfin treatment enhanced and reduced Atg5 promoter activity, respectively, whereas mutation of the TEAD1-binding site abrogated the effects of YAP overexpression and verteporfin treatment (Figure 2G, 2H). In addition, we analyzed the correlation between YAP and Atg5 expression in the TCGA PAAD (pancreatic adenocarcinoma) database and found a positive correlation between YAP and Atg5 mRNA expression levels (R=0.63, *p*<0.001) (Figure 2I). As expected, Atg5 mRNA expression was increased in YAP overexpressing PDAC cells and decreased in YAP knockdown PDAC cells (Supplementary Figure S4A and S4B). In addition, the protein expression of ATG5 also changed with YAP overexpression or knockdown accordingly (Figure 2J, 2K). Collectively, these findings strongly suggest that YAP promotes Atg5 transcription through the TEAD1-binding site. In addition, we also validated the effect of Atg5 on autophagy induction by YAP. We found that Atg5 knockdown inhibited YAP-induced autophagy activation (Figure 2L), while ectopic expression of Atg5 rescued YAP depletion-mediated autophagy inhibition (Figure 2M). These results suggest that YAP-induced autophagy is Atg5 dependent.

### Autophagy negatively regulates YAP through autophagic degradation

**Figure 3:**
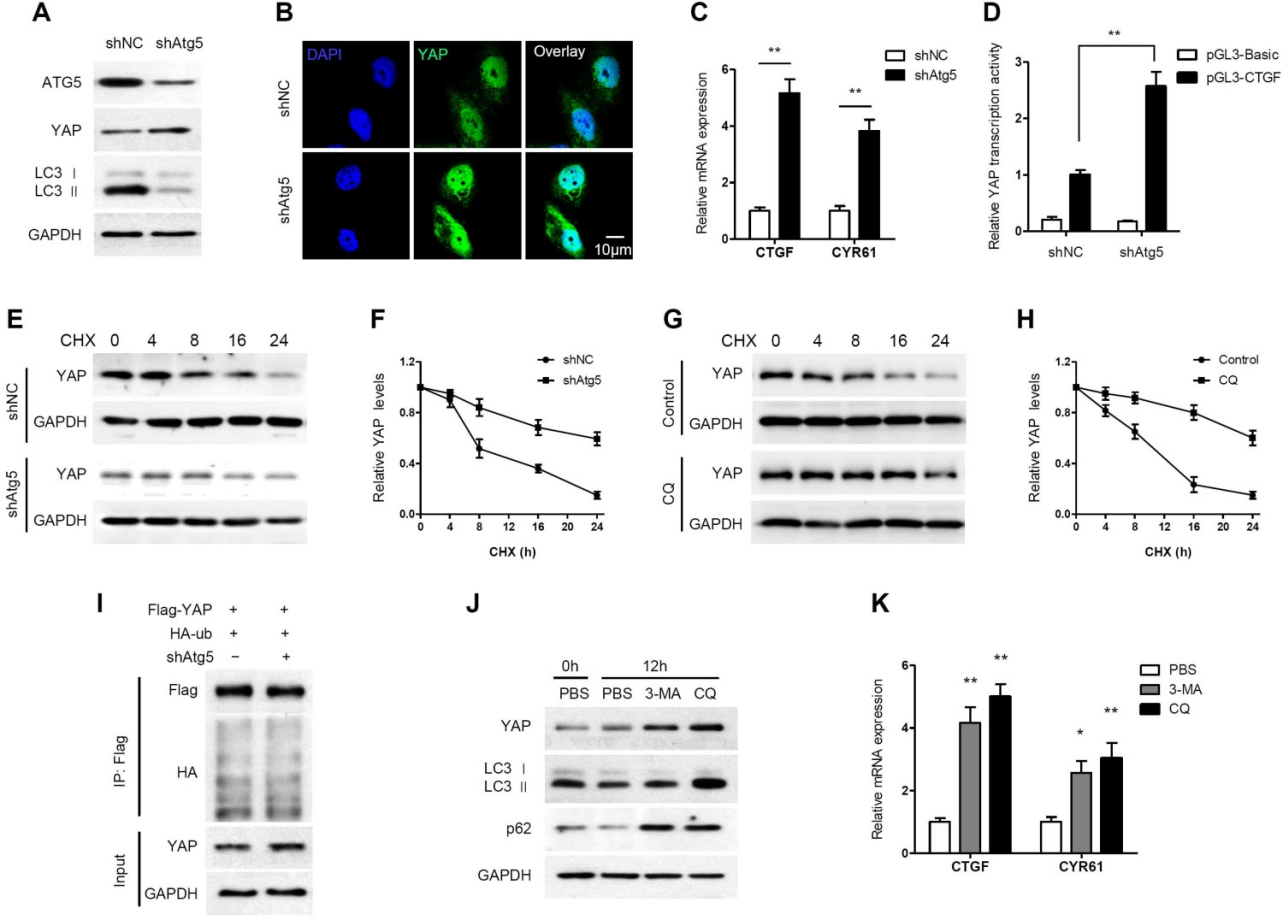
** Autophagy negatively regulates YAP through autophagic degradation. (A)** AsPC-1 cells were transfected with shAtg5 and a negative control, and the protein expression levels were analyzed by immunoblotting. **(B)** Immunofluorescence analysis of YAP in AsPC-1 cells transfected with shAtg5 or a negative control. **(C)** RT-qPCR analysis of the mRNA levels of YAP target genes in AsPC-1 cells transfected with shAtg5 and a negative control. **(D)** Luciferase reporter assay to evaluate the activity of YAP from AsPC-1 cells transfected with shAtg5 and a negative control. **(E, F)** AsPC-1 cells were transfected with shAtg5 or a negative control, and YAP was detected by immunoblotting after CHX (20 μM) incubation. **(G, H)** AsPC-1 cells were treated with or without CQ (100 mM) and YAP was detected by immunoblotting after CHX (20 μM) incubation. **(I)** AsPC-1 cells were transfected with Flag-YAP and HA-ubiquitin (HA-Ub) plasmids and shAtg5 or a negative control. The cell lysates were subjected to immunoprecipitation. **(J)** AsPC-1 cells were treated with CQ (100 mM), 3-MA (5 mM) or PBS only, and the protein expression levels were analyzed by immunoblotting. **(K)** RT-qPCR analysis of the mRNA levels of CTGF and CRY61 in AsPC-1 cells treated with CQ (100 mM), 3-MA (5 mM) or PBS. Data are shown as the mean ± SEM from three independent experiments. Student's *t* test. *p<0.05, **p<0.01, ***p<0.001.

Generally, when the Hippo pathway is activated, YAP is phosphorylated and degraded through the βTrCP-mediated proteasomal pathway. However, a novel mechanism of YAP degradation has been recently identified. It was found that YAP is an autophagy substrate and an essential downstream mediator of hepatic differentiation and carcinogenesis in autophagy-deficient livers 36. Therefore, we investigated the effect of autophagy on YAP in PDAC cells. Atg5 knockdown increased YAP accumulation and nuclear localization in AsPC-1 cells (Figure 3A, 3B). In addition, Atg5 knockdown remarkably increased the mRNA expression levels of the YAP targets CTGF and CYR61 (Figure 3C). We also examined the impact of autophagy on the transcriptional activity of YAP using a luciferase assay. The transcriptional activity of YAP was significantly enhanced when autophagy was inhibited by Atg5 knockdown (Figure 3D). However, YAP mRNA levels did not change significantly when Atg5 was knocked down or overexpressed (Supplementary Figure S5A and S5B). It suggests that ATG5 regulation of YAP is independent of transcriptional regulation.

Moreover, the degradation of YAP was detected by cycloheximide (CHX) chase assay. The half-life of YAP was increased in Atg5-knockdown cells (Figure 3E, 3F). Similarly, the half-life of YAP was increased in CQ-treated cells (Figure 3G, 3H). To investigate whether ubiquitin is involved in autophagy-mediated YAP degradation, we performed a co-IP assay and found no significant change in the ubiquitination level of YAP after Atg5 knockdown (Figure 3I). This indicates that autophagy-mediated YAP degradation is ubiquitin-independent.

In contrast to CQ, 3-methyladenine (3-MA) inhibits autophagy by blocking autophagosome formation via the inhibition of class III PI3K. As expected, LC3 II levels were decreased by treatment with 3-MA, whereas CQ increased the accumulation of LC3 II (Figure 3J). However, both 3-MA and CQ, inhibiting autophagosome formation and inhibiting autophagosome degradation respectively, led to increased YAP protein levels (Figure 3J). Moreover, both CQ and 3-MA significantly enhanced CTGF and CRY61 transcription compared to that observed in the control (Figure 3K). In conclusion, these results suggest that YAP can be degraded through the autophagy pathway in PDAC cells, and that there is a negative feedback loop between autophagy and YAP.

### Autophagy weakened the role of YAP in promoting PDAC cell proliferation and migration

**Figure 4:**
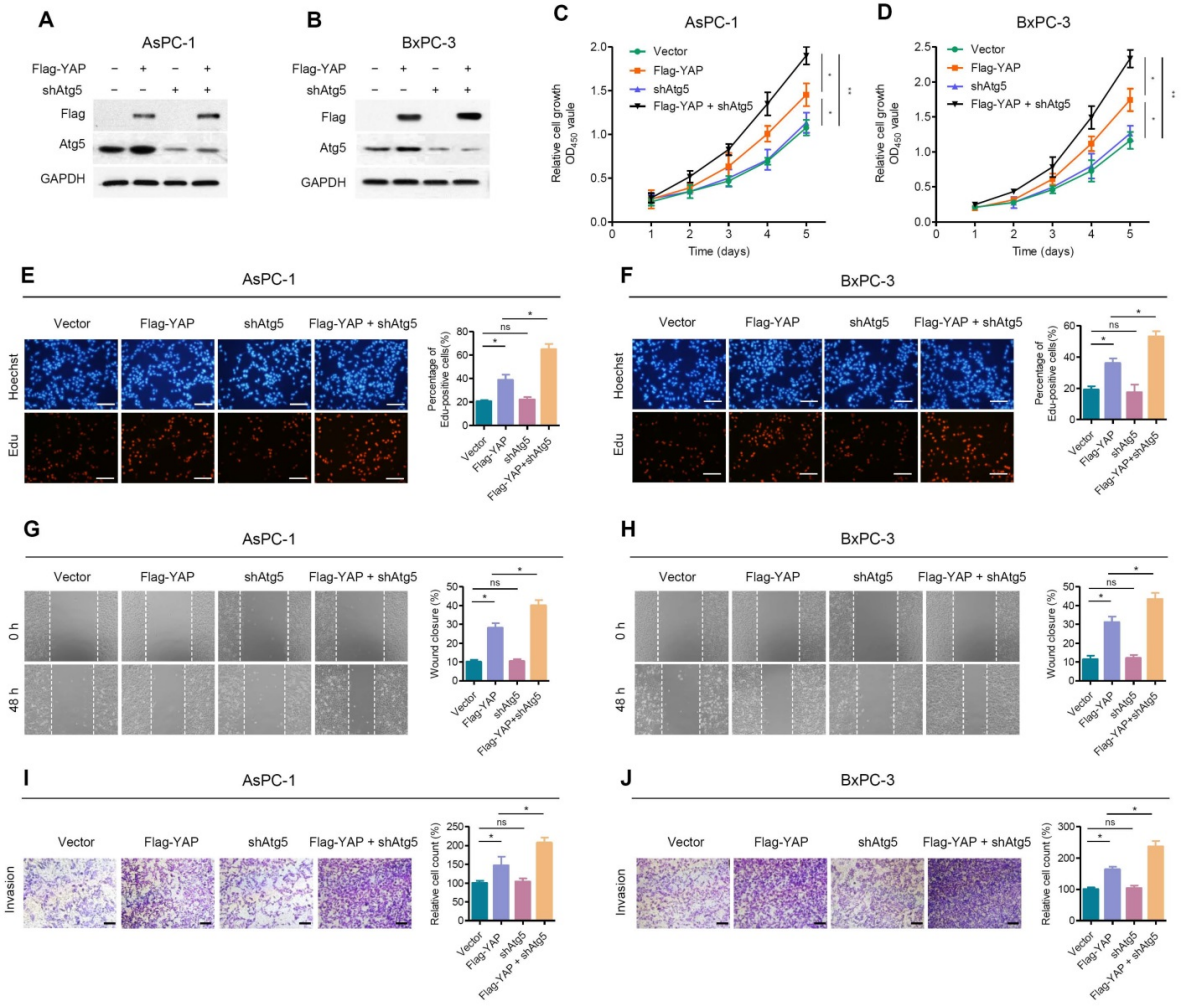
**Inhibition of autophagy attenuates the effect of YAP on pancreatic cancer cells. (A, B)** AsPC-1 and BxPC-3 cells were transfected with shAtg5 and Flag-YAP simultaneously or separately. Immunoblot showing the expression levels of the indicated proteins. **(C, D)** Cell viability was determined using the CCK-8 assay in AsPC-1 and BxPC-3 cells transfected with the indicated vectors. **(E, F)** EdU incorporation assays were performed in AsPC-1 and BxPC-3 cells transfected with the indicated vectors. Scale bar, 100 µm. **(G, H)** Wound healing migration assays in AsPC-1 and BxPC-3 cells transfected with the indicated vectors. **(I, J)** Transwell invasion assays in AsPC-1 and BxPC-3 cells transfected with indicated vectors. Scale bar, 200 µm. Data are shown as the mean ± SEM from three independent experiments. Student's *t* test. *p<0.05, **p<0.01, ***p<0.001. ns, no significant.

Due to the negative feedback between autophagy and YAP, we further investigated the effect of autophagy on YAP-promoted malignant progression of PDAC cells. We first examined the transfection efficiency of shAtg5 and Flag-YAP in AsPC-1 and BxPC-3 cells (Figure 4A, 4B). Atg5 depletion enhanced YAP ectopic overexpression-induced cell proliferation, as determined by CCK-8 and EdU cell proliferation assays (Figure 4C-F). Moreover, wound healing (Figure 4G, 4H) and Transwell invasion assays (Figure 4I, 4J) indicated that Atg5 knockdown significantly enhanced the promoting effect of YAP ectopic overexpression on the migration and invasion of PDAC cells. It is worth noting that Atg5 depletion alone did not significantly affect the proliferation and migration of PDAC cells.

The possible reason is that, at the basal level, the promoting effect of basal autophagy on PDAC cells counteracts its negative feedback regulation of YAP. However, in the case of YAP overexpression, the promoting effect of autophagy itself on PDAC was almost negligible, while autophagy inhibition led to the accumulation of YAP and further promoted the activity of YAP. Therefore, Atg5 depletion itself has a weak effect but can enhance the effect of YAP on PDAC cells.

### Rapamycin enhances the antitumor effect of verteporfin in PDAC

**Figure 5:**
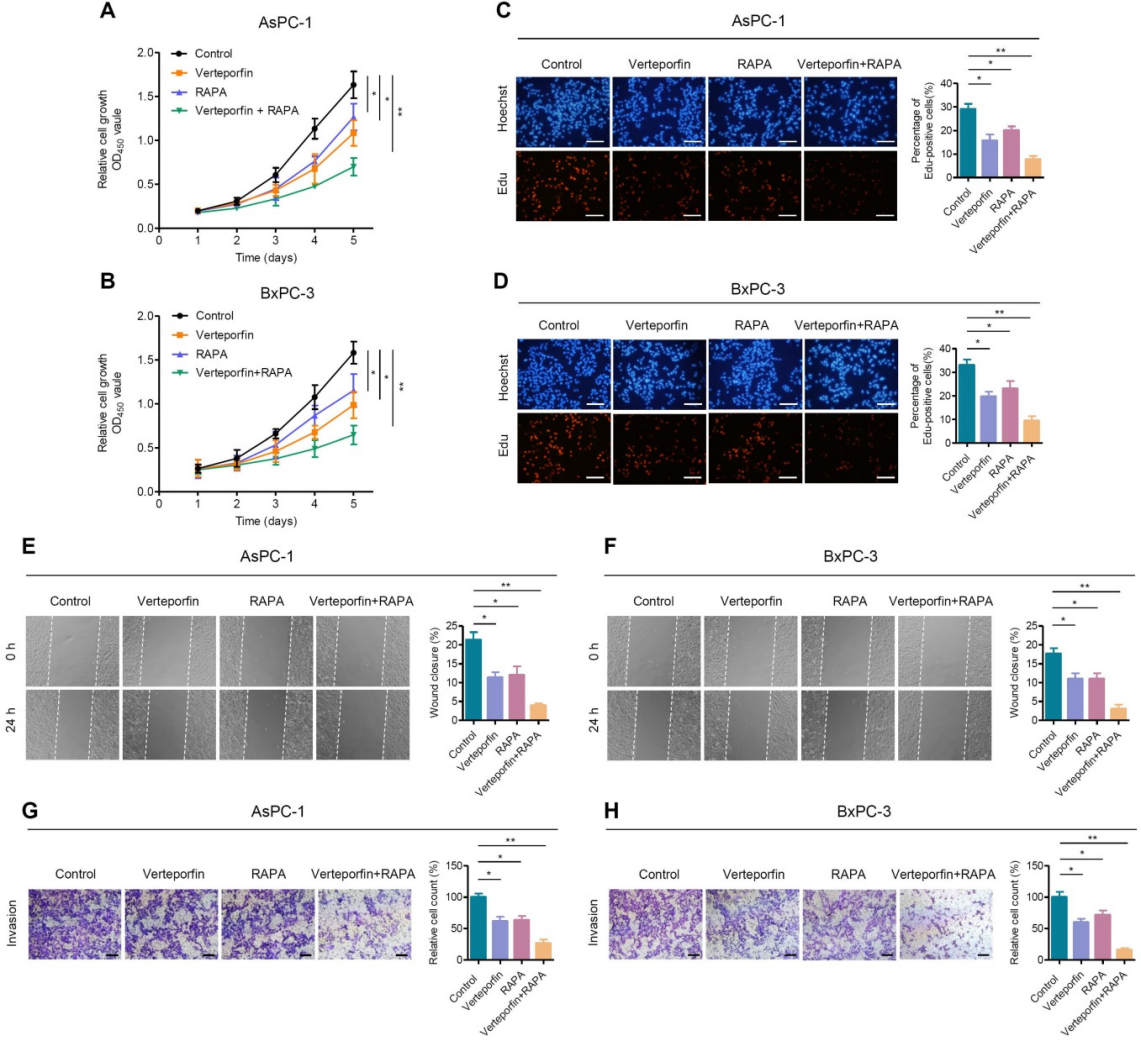
** Rapamycin enhances the effect of verteporfin in suppressing pancreatic cancer cell proliferation, growth and motility. (A, B)** AsPC-1 and BxPC-1 cells were treated with verteporfin and rapamycin simultaneously or separately, and cell viability was determined by CCK-8 assay. **(C, D)** EdU incorporation assays were performed in AsPC-1 and BxPC-3 cells with the indicated treatment. Scale bar, 100 µm. **(E, F)** Wound healing migration assays in AsPC-1 and BxPC-3 cells with the indicated treatments. **(G, H)** Transwell invasion assays in AsPC-1 and BxPC-3 cells with the indicated treatments. Scale bar, 200 µm. Data are shown as the mean ± SEM from three independent experiments. Student's *t* test. *p<0.05, **p<0.01, ***p<0.001.

**Figure 6:**
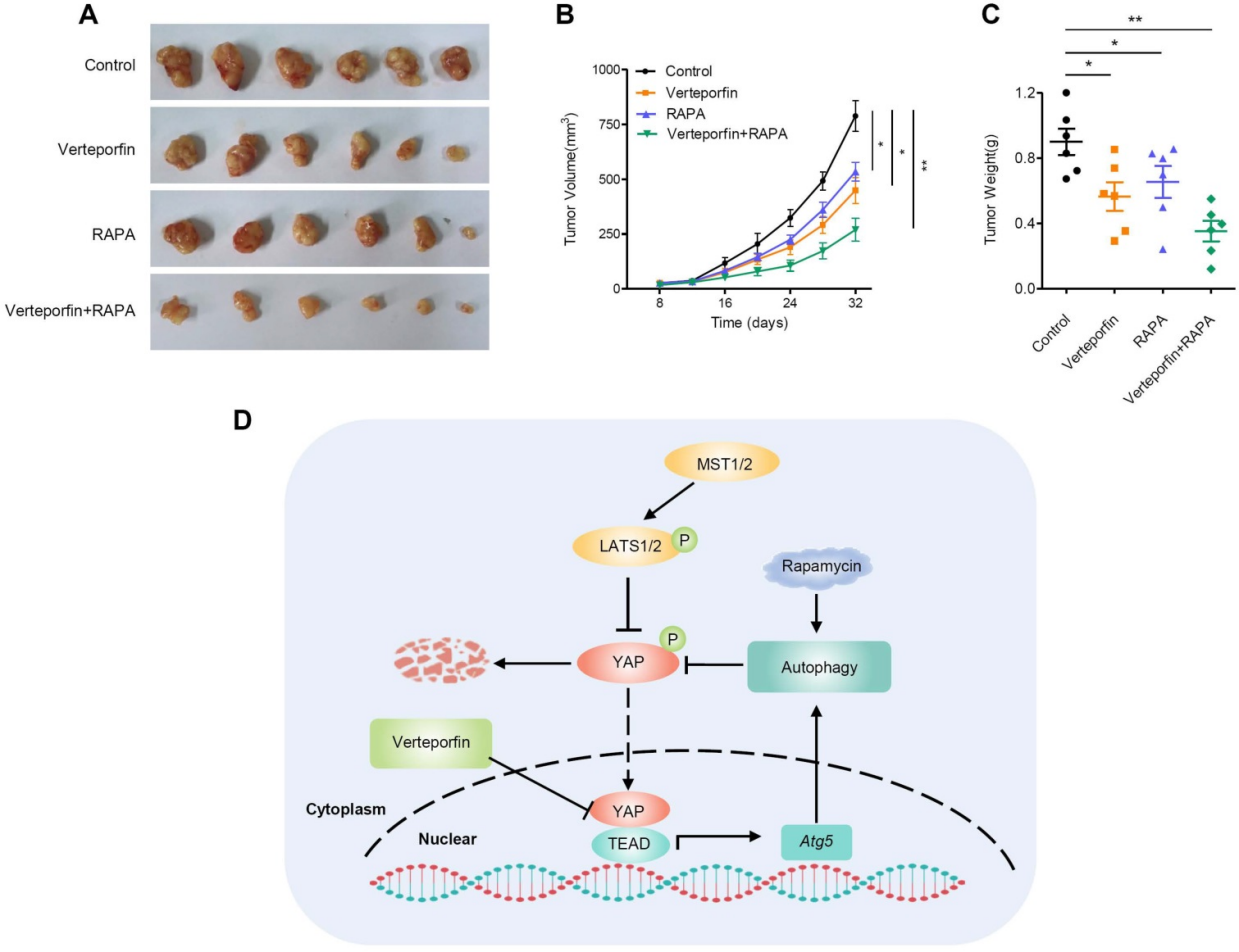
** Rapamycin enhances the antitumor effect of verteporfin. (A-C)** Combination effect of verteporfin and rapamycin in established pancreatic cancer xenografts (n = 6 per group). Schematic representation (A), growth curves (B), and tumor weights were measured at the end of the experiment (C). The values represent the mean ± SEM. Student's *t* test. *p<0.05, **p<0.01, ***p<0.001. (D) A proposed model for the crosstalk between YAP and autophagy in promoting pancreatic cancer progression.

Rapamycin, an mTORC1-dependent autophagy activator, has been approved by the FDA for cancer therapy and immunosuppression 45. We further evaluated the effects of autophagy activation and YAP inhibition, mediated by rapamycin and verteporfin respectively, on PDAC cells. We found that, the combination of verteporfin and rapamycin significantly inhibited the proliferation of AsPC-1 and BxPC-3 cells compared with that observed with either verteporfin or rapamycin treatment alone (Figure 5A-D). Similar effects on cell migration and invasion were also observed in PDAC cells (Figure 5E-H). We further evaluated the combinational antitumor effect *in vivo* by establishing a subcutaneous pancreatic cancer xenograft model in nude mice injected with AsPC-1 cells. We found that the combination of verteporfin and rapamycin profoundly inhibited tumor growth in mice (Figure 6A-C). These results imply that activation of autophagy can significantly enhance the antitumor activity of verteporfin and that targeting the YAP-autophagy circuit may be a potential therapeutic strategy for pancreatic cancer.

## Discussion

Cell-intrinsic negative feedback loops are important to ensure proper physiological regulation and homeostasis of cells. The findings presented here support the existence of a feedback loop consisting of YAP and autophagy that regulates PDAC progression. YAP activates autophagy via TEAD1-mediated transcription of Atg5. Moreover, autophagy negatively regulates YAP through autophagic degradation. The complex regulatory circuit of YAP and autophagy provides feedback regulation of YAP and thus ensures tissue homeostasis. However, aberrant hyperactivation of YAP disruptes this feedback loop and promotes the malignant progression of PDAC. Autophagy activation and YAP inhibition collectively suppresses the progression of PDAC (Figure 6D).

YAP is essential for cancer initiation and progression but dispensable for normal homeostasis in adult organs, making it an attractive target for cancer therapy 17. Since YAP association with TEADs is essential for YAP transcriptional effects in most cellular contexts 46, blocking the YAP-TEAD interaction represents one of the most promising strategies for realizing anti-YAP therapy. Verteporfin can disrupt the interaction between YAP and TEAD, thus abrogating YAP-induced transcription 44. As a clinical photosensitizer for the treatment of macular degeneration, verteporfin has also been shown to be safe in clinical trials 47.

Autophagy is a mechanism by which cellular material is delivered to lysosomes for degradation, leading to the basal turnover of cell components and providing energy and macromolecular precursors. Autophagy has opposing, context-dependent roles in cancer. Although sometimes controversial, targeting autophagy has been proposed as a potential therapeutic strategy for cancer 48. Rapamycin, an mTORC1-dependent autophagy activator, is an FDA-approved drug that is safe for human use. The antitumor effect of rapamycin has been reported in different types of cancer 49, 50. In our study, inhibition of YAP by verteporfin or activation of autophagy by rapamycin suppressed the malignant phenotype of PDAC to some extent both* in vitro* and* in vivo*. However, the combination of verteporfin and rapamycin was more robust in suppressing PDAC progression.

In conclusion, our findings reveal the existence of a YAP-autophagy feedback loop that regulates PDAC progression. YAP transcriptionally activats Atg5 via interaction with TEAD1, which in turn initiates negative feedback regulation through autophagic degradation of YAP. However, in PDAC, aberrant hyperactivation of YAP disrupts this homeostasis, inducing YAP-autophagy circuit imbalance and subsequent malignant cancer progression. Moreover, our study indicates that targeting the YAP-autophagy signaling circuit may represent a novel therapeutic strategy for pancreatic cancer.

## Supplementary Material

Supplementary figures and tables.

## Abbreviations

ChIP: chromatin immunoprecipitation
CQ: chloroquine
EGFR: epidermal growth factor receptor
EMT: epithelial-to-mesenchymal transition
IHC: immunohistochemical
PDAC: pancreatic ductal adenocarcinoma
RT-qPCR: Real-time quantitative polymerase chain reaction
YAP: Yes-associated protein
3-MA: 3-Methyladenine
