# Supplementary material for: Autophagy-mediated negative feedback attenuates the oncogenic activity of YAP in pancreatic cancer
Source: Int J Biol Sci. 2021 Aug 21;17(13):3634–45. doi: 10.7150/ijbs.61795 (PMC8416727; doi:10.7150/ijbs.61795)
Supplement: Supplementary file 1 — Supplementary figures and tables. [file ijbsv17p3634s1.pdf]

Supplementary Tables and Figures

Table S1. Primer sequences for RT-PCR.

Gene	Primer sequence
CTGF	F: 5’-AGGAGTGGGTGTGTGACGA-3’<br>R: 5’-CCAGGCAGTTGGCTCTAATC-3’
Atg5	F: 5’-CAGACAACGACTGAAAGACCT-3’<br>R: 5’-CAGGATCAATAGCAGAAGGACA-3’
GAPDH	F: 5’-ACTCCTCCACCTTTGACGC-3’<br>R: 5’-GAGGGGAGATTCAGTGTGGTG-3’

Table S2. Primer sequences for RT-PCR in ChIP assay.

Gene	Primer sequence
CTGF	F: 5’-CTCCCTCAGGCTGCATGTTC-3’<br>R: 5’-CTGGGATGCAAAGGGGGTTC-3’
Atg5	F: 5’-CTGGGAAGGCAATGCACCTT-3’<br>R: 5’-ACAGTGTCCCCAGTAAACAGC-3’
GAPDH	F: 5’-GCAGAGGGCGTGAGCTATG-3’<br>R: 5’-GTCTTCTCCCCGCAAAGAGG-3’

Table S3. Predicted binding sites of TEAD1 in the Atg5 promoter.

Score	Relative score	Start[a]	End	Predicted site sequence
12.8296	0.9865	-1071	-1062	CACATTCCTA
12.0135	0.8968	-1071	-1060	CACATTCCTATC
7.42914	0.8752	-1045	-1036	TACATACTGA
5.99244	0.8456	-1396	-1387	TAGATTCTAC
5.99244	0.8456	-1382	-1373	TAGATTCTAC

[a] position relative to the transcription initiation site (defined as the +1 position) of Atg5, e.g., -1071 means an element located 1071 nucleotides upstream of Atg5 transcription start site.

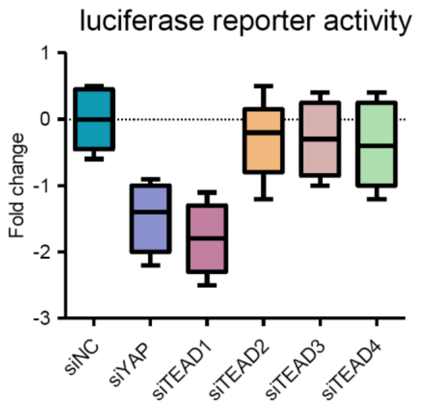

Figure S1. Expression change of a YAP/TEAD responsive luciferase reporter upon siRNA-mediated knockdown of YAP or TEADs normalized to a negative control siRNA in AsPC-1 cells.

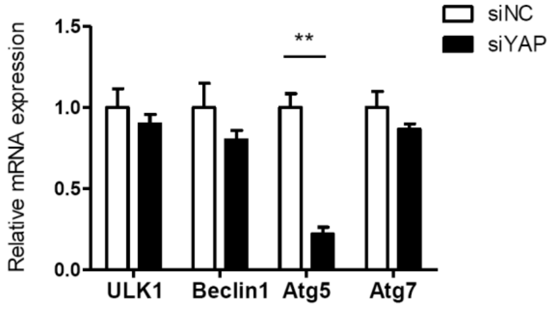

Figure S2. RT-qPCR analysis of the mRNA levels of ULK1,Beclin1, Atg5 and Atg7 in AsPC-1 cells treated with siYAP or a negative control siRNA. Data are shown as mean ± SEM from three independent experiments. Student's t test. ***p*<0.01, ****p*<0.001.

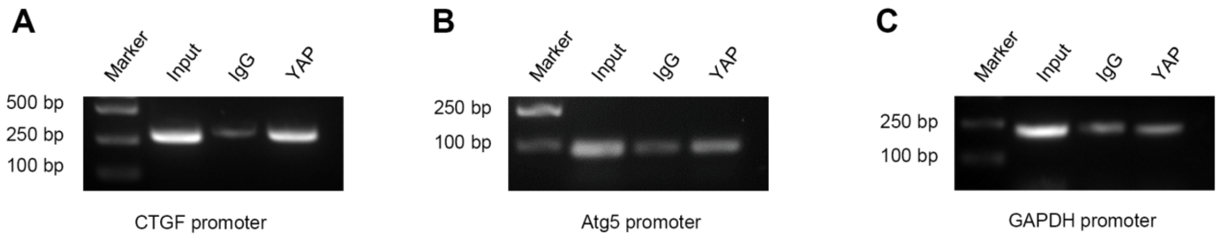

Figure S3. ChIP assays with YAP antibody or control IgG were performed on chromatin derived from AsPC-1 cells. Primers of CTGF, Atg5 and GAPDH promoter were used for PCR amplification.

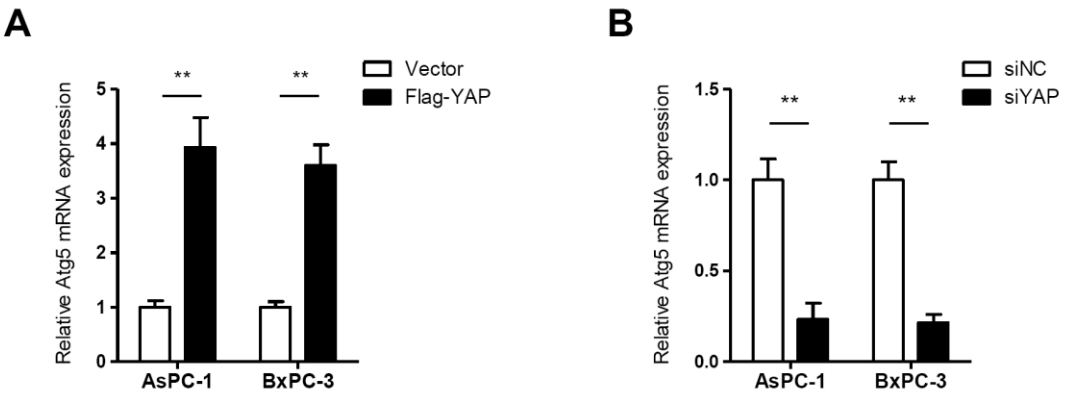

Figure S4. RT-qPCR analysis of Atg5 mRNA expression in AsPC-1 and BxPC-3 cells with YAP overexpression or knockdown.

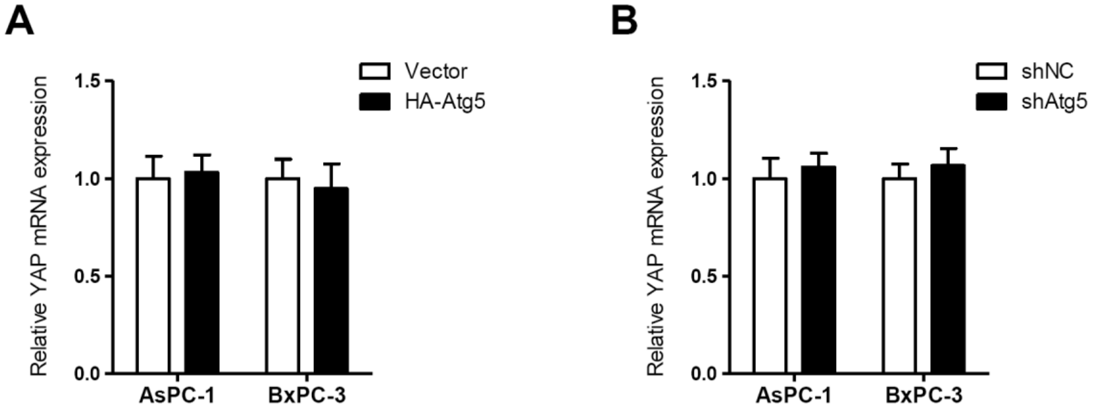

Figure S5. RT-qPCR analysis of YAP mRNA expression in AsPC-1 and BxPC-3 cells with Atg5 overexpression or knockdown.
